# Potentiation of the antimycobacterial activity of bedaquiline, clofazimine, and doxycycline against *Mycobacterium smegmatis *by several natural product-based compounds is putatively via efflux inhibition

**DOI:** 10.12688/openresafrica.16071.1

**Published:** 2025-10-29

**Authors:** Robi Chacha, Caroline Maina, Edwin Murungi, Eric Guantai, Mathew P. Ngugi, Ephantus Ndirangu, James Mugo, Sospeter Ngoci Njeru, Elizabeth Kigondu

**Affiliations:** 1 Centre for Respiratory Diseases Research, Kenya Medical Research Institute, Nairobi, Nairobi County, Kenya; 2 Department of Biochemistry, Microbiology and Biotechnology, Kenyatta University School of Pure and Applied Sciences, Nairobi, Nairobi County, Kenya; 3 Centre for Traditional Medicine and Drug Research, Kenya Medical Research Institute, Nairobi, Nairobi County, Kenya; 4 Department of Chemistry, University of Cape Town Department of Chemistry, Rondebosch, Western Cape, South Africa; 5 Department of Medical Biochemistry, Kisii University College School of Health Sciences, Kisii, Kisii County, Kenya; 6 Department of Pharmacology, Clinical Pharmacy and Pharmacy Practice, Faculty of Health Sciences, University of Nairobi, Nairobi, Nairobi County, Kenya; 7 Department of Chemistry, Kenyatta University Department of Chemistry, Nairobi, Nairobi County, Kenya; 8 Centre for Community Driven Research, Kenya Medical Research Institute, Kirinyaga, Kirinyaga County, Kenya

**Keywords:** Efflux pumps, Mycobacterium smegmatis, Mycobacterium tuberculosis, molecular docking, molecular dynamics, efflux inhibition, natural products.

## Abstract

**Background:**

*Mycobacterium tuberculosis* ( *Mtb*), the causative agent of tuberculosis (TB), is the leading cause of death due to a single infectious pathogen globally. The increasing prevalence of drug-resistant *Mtb* strains underscores the pressing need for the development of new antimycobacterial drugs with novel mechanisms of action. Targeting pathogen drug efflux, a key antimycobacterial drug resistance mechanism, is an attractive, viable strategy for the development of new TB therapeutics.

**Methods:**

In this study, we utilised *Mycobacterium smegmatis* ( *Msm*), a non-pathogenic *Mtb* surrogate, to delineate the ability of natural-product based compounds to augment the efficacy of bedaquiline (BDQ), clofazimine (CFZ) and doxycycline (DOX), probably via efflux inhibition (EI). Literature reporting the plant sources of the known efflux inhibitors (EIs) reserpine (RES), berberine (BER) and piperine (PIP) was scoped and additional compounds, (+)-lyoniresinol-3-Alpha-O-Beta-D-glucopyranoside (LYO-3) and lyoniresinol (LYO), isolated from the same plant species were chosen for testing. *In vitro* screening of the selected compounds was performed using the two-dimensional (2-D) checkerboard assay in which each likely efflux disruptor was tested in combination with BDQ, CFZ and DOX against *Msm* and the effect of the combinations ascertained. Thereafter, compounds that exhibited probable EI activity were docked onto potential targets namely MSMEG_5187, a *Msm* homologue of *Mtb* Rv1258c efflux pump (EP) and MmpL5.

**Results:**

Molecular docking revealed that the EIs avidly bound to the EPs with docking scores of <-7 kcal/mol while the checkerboard combination assays demonstrated strong synergistic interactions for BDQ plus BER, LYO-3 and LYO.

**Conclusion:**

These results point to a probable disruption of the *Msm* efflux system by *Berberis species* derivatives.

## Introduction

Tuberculosis (TB), an infectious disease caused by *Mtb* that primarily affects the pulmonary system, is the leading cause of mortality by a single infectious agent globally. Of the estimated 10.8 million new TB cases and 1.25 million deaths reported worldwide in 2023, the majority (85%) of the cases and fatalities occurred in the low and middle-income countries of Africa and Asia ( *Global Tuberculosis Report 2024*, 2024). The current WHO-recommended TB treatment is lengthy (typically a 4–6 month combination regimen) and has a major pitfall of patient non-compliance, which may contribute to the development of drug resistance due to underdosing. The regimen is underpinned by four first-line agents, namely isoniazid (INH), rifampicin (RIF), ethambutol (EMB), and pyrazinamide (PZA), that are used together with several second-line drugs, including BDQ, linezolid, and ( Saukkonen *et al.*, 2025; Singh *et al.*, 2020). However, the efficacy of various TB combination regimens is increasingly being hindered by the growing number of multidrug-resistant (MDR) and extensively drug-resistant (XDR) *Mtb* clinical isolates ( Alsayed & Gunosewoyo, 2023). Thus, there is a pressing need for the discovery of new antibiotics with novel mechanisms of action and potentiators of current TB drugs.

An attractive strategy for the development of potentiators for anti-TB drugs is the targeting of drug efflux, a key mechanism for intrinsic *Mtb* drug resistance ( Laws *et al.*, 2022; Menichini *et al.*, 2020; Rindi, 2020). Coupled with the impermeability of the mycobacterial cell, enhanced drug efflux, which occurs as an early stress response to drug exposure, likely triggers the development of stable and heritable drug resistance ( Campolattano *et al.*, 2023). Indeed, drug efflux has been implicated in *Mtb* resistance to BDQ, a recently approved agent for MDR and XDR TB infections ( Derendinger *et al.*, 2023). Thus, disrupting *Mtb* efflux pumps (EPs) would plausibly reverse resistance by augmenting the activity of current anti-TB drugs and with the consequence of shortening the treatment duration ( Ghosh *et al.*, 2021). Of the more than 50 reported *Mtb* EPs, MmpS5-MmpL5, Rv1258c and Rv1819c have been linked with drug efflux in MDR and XDR TB infections ( Rodriguez-Takeuchi *et al.*, 2019). 

The MmpS5-MmpL5 efflux system is one of the best characterized. MmpL5 serves as the main transmembrane transporter, while MmpS5 acts as an accessory stabilizing protein. Expression of the mmpS5-mmpL5 operon is repressed by Rv0678, a MarR-family regulator. Mutations in Rv0678, commonly selected under drug pressure, lead to pump overexpression and enhanced drug efflux. This system has been implicated in the extrusion of BDQ, clofazimine, azoles, and tetracyclines ( Laws *et al.*, 2022; Ma *et al.*, 2020). Importantly, the overexpression of MmpS5–MmpL5 reduces intracellular BDQ concentrations and consequently diminishes inhibition of ATP synthase, illustrating the direct interplay between efflux-mediated resistance and energy metabolism in *Mycobacteria*.

The Rv1258c (Tap) is a member of the major facilitator superfamily (MFS). It is a single-component transporter that uses the proton motive force to export drugs. It has been associated with resistance to a wide array of first- and second-line agents, including RIF, INH, PZA, EMB, aminoglycosides, tetracyclines, and spectinomycin ( Liu *et al.*, 2019). Overexpression of Rv1258c, often mediated by WhiB7-dependent regulation, enhances its efflux capacity and has been linked to multidrug resistance in clinical isolates ( Sharma *et al.*, 2010).

A third system, Rv1819c, belongs to the ATP-binding cassette (ABC) transporter family. Unlike Rv1258c, it couples ATP hydrolysis to drug efflux and features a large translocation channel capable of accommodating diverse substrates ( Rempel *et al.*, 2020). Overexpression or mutation of Rv1819c has been associated with reduced susceptibility to BDQ and azoles ( Andries *et al.*, 2014). Together, MmpS5–MmpL5, Rv1258c, and Rv1819c highlight the mechanistic diversity of efflux-mediated resistance in *Mtb* and underscore the therapeutic potential of targeting these systems with EIs.

Natural products (NPs) have been demonstrated to be effective EIs ( Beutler, 2019; Ramalingam *et al.*, 2024). For instance, RES has been shown to block tetracycline extrusion in *Bacillus subtilis* ( Shaheen *et al.*, 2019a), while PIP disrupts *Mtb* Rv1258c ( Kapp *et al.*, 2021). Moreover, BER has activity against *Escherichia coli* MdfA ( Li & Ge, 2023a). Building on this evidence, our study explored other secondary metabolites isolated from plant sources of known EIs to uncover new NPs that can be explored as EIs in mycobacteria. Altogether, this study has uncovered NP-based compounds exhibiting synergistic activity when co-administered with BDQ, CFZ, and DOX plausibly by blocking MmpS5-MmpL5 and MSMEG_5187 EPs.

## Materials and methods

### Chemicals and bacterial strains

Berberine chloride (BER, Cat. No. B3251), reserpine (RES, Cat. No. R0875), piperine (PIP, Cat. No. P49007), bedaquiline fumarate (BDQ, Cat. No. SBR00060-10MG), doxycycline hyclate (DOX, Cat. No. D9891), clofazimine (CFZ, Cat. No. C8895), dimethyl sulfoxide (DMSO, Cat. No. D2650), Middlebrook Oleic Albumin Dextrose Catalase Growth Supplement (OADC, Cat. No. M0678), Glycerol (Cat. No. G5516), Middlebrook 7H9 Broth Base (Cat. No. M0178), and Resazurin sodium salt (Cat. No. R7017) were obtained from Sigma-Aldrich Merck KGaA (Germany), while lyorisenol-3-alpha (LYO-3) and lyorisenol (LYO) were purchased from ChromaDex (USA). The non-pathogenic *Msm* strain ATCC 607 was sourced from frozen glycerol stocks maintained at the Centre for Respiratory Diseases Research, Kenya Medical Research Institute (CRDR-KEMRI). Cytotoxicity assays were performed using African green monkey kidney epithelial cells (Vero E6, ATCC® CRL-1586™), which were seeded from passaged stocks maintained at the Centre for Traditional Medicine and Drug Research, Kenya Medical Research Institute (CTMDR-KEMRI).

### Identification of putative efflux inhibitors

To determine the probable EIs for experimental and computational evaluation, a comprehensive review of peer-reviewed NPs literature was undertaken to identify plant-derived EIs previously demonstrated to enhance the antimycobacterial activity of antibacterials. Following the retrieval and evaluation of several relevant publications reporting the phytochemical origins of EIs with synergistic activity in combination with standard antibacterial agents, the source plant species were documented ( Mokhber-Dezfuli *et al.*, 2014; Puk & Guz, 2022; Seukep *et al.*, 2020). Subsequently, phytochemicals isolated from the identified plant species were ascertained and potential EIs parsed based on structural similarity to compounds reported to inhibit efflux. To broaden the number of potential EIs, a search of chemical and structural analogs to the identified compounds in public databases including ZINC, PubChem and ChemSpider was performed. Specifically, to constrain the search to relevant candidates, database queries utilized structural similarity and substructure search parameters. Following physicochemical profiling, the antimicrobial activity of selected compounds was determined using *in vitro* assays.

### Anti-mycobacterial screening

The Resazurin Microtiter Assay (REMA) was utilized to qualitatively determine the minimum inhibition concentration (MIC) of BDQ, CFZ, DOX, RES, PIP, BER, LYO, and LYO-3 against *Msm* as previously described by Sarker *et al.*, 2007. The visual MIC was defined as the lowest concentration with no bacterial pellet and no color change of resazurin from blue to pink. The test compounds were prepared at a stock concentration of 10mM in 100% DMSO (Sigma Aldrich). Two-fold serial dilutions were prepared in 7H9 broth supplemented with 10% OADC, 0.02% Glycerol, and 0.05% Tw80 (7H9_OADC_GLY_TW) broth medium in 96-well round-bottom plates, starting with the addition of 50µl of medium to each well, followed by 2.5µl of the test compounds. A range of concentrations was evaluated for each compound. Each test plate included a maximum inhibition control (wells with media, 0.5µg/ml of RIF and bacteria) and a minimum inhibition control (wells containing only media and bacteria). The culture of *Msm* was grown in 7H9_ADC_GLY_TW to an optical density (OD _600_) of 0.5–0.7, then reconstituted to an OD _600_ of 0.001 in medium. To each well, 50 µl of the diluted culture was added to give a final volume of 100 µl per well (50 µl of medium and 50 µl of diluted culture). Each compound was tested in three technical replicates, and the plates were incubated for 48 h at 37 °C and visually scored. Thereafter, one-tenth of the assay volume of resazurin (0.2 mg/ml prepared in PBS) was added to each well, and the plates were re-incubated for an additional 8 hrs.

### Drug combination (checkerboard) assay

A two-dimensional (2-D) checkerboard assay incorporating resazurin was performed to establish the interaction between each drug in combination with the putative EIs against *Msm* in a 96-well plate. The fractional inhibitory concentration index (FICI) and the fractional inhibitory concentration (FIC) for each combination were calculated as previously described ( Ramón-García *et al.*, 2011). The FIC for each compound was calculated as follows: FIC _A_ = (MIC of compound A in the presence of compound B) / (MIC of compound A alone), where FIC _A_ represents the FIC of compound A. The FIC for compound B (FIC _B_) was calculated similarly. The overall FICI is the sum of FIC _A_ and FIC _B_. The potential interactions between each anti-TB drug and EI were classified based on the FICI values as follows: synergy: FICI ≤ 0.5, antagonism: FICI > 4.0, no interaction: FICI 0.5-4.0. All experiments were performed in three technical replicates and expressed as an average of the three values.

### Cytotoxicity assay

To determine the cytotoxicity of the compounds, Vero E6 cells (derived from the African green monkey kidney cells) were used and the MTT assay was performed as described by Mosmann (1983). The Vero E6 cells were sourced from the American Type Culture Collection (ATCC, Manassas, Virginia, USA). The cells were maintained in Eagle’s Minimum Essential Medium (EMEM) supplemented with 10% fetal bovine serum (FBS; GIBCO, USA), 1.5% sodium hydrogen carbonate (LOBA Chemie, India), 1% GlutaMAX (GIBCO, USA), 1% penicillin–streptomycin (GIBCO, USA), and 1% HEPES buffer (Gold Biotechnology, USA). They were incubated at 37°C in a humidified incubator (5% CO _2_, 98% relative humidity; Panasonic, Japan) until 80% confluency was achieved, after which they were sub-cultured for cytotoxicity evaluation.

At 80% confluency, cells were washed with phosphate-buffered saline (PBS; Sigma-Aldrich, USA) and detached using 0.25% trypsin–EDTA (Solarbio, China). Trypsin activity was neutralized by adding supplemented EMEM. Cells were stained with trypan blue (Loba Chemie, India) and counted using a hemocytometer, and the cell suspensions were adjusted with supplemented EMEM to a final density of 1 × 10 ^5^ cells/mL. Vero E6 cells were then seeded at 1 × 10 ^4^ cells/well into 96-well plates (Greiner Bio-One, Austria) and incubated overnight at 37 °C in a humidified incubator (5% CO _2_, 98% relative humidity).

After 24 hrs, the culture medium was replaced with fresh treatment medium with dose dilutions. Cells were incubated for 48hrs at 37 °C and 5% CO _2_, 98% relative humidity. After 48 hrs incubation, the media was removed, and the cells were rinsed with PBS (Sigma-Aldrich, USA). Fresh EMEM supplemented with MTT dye (5 mg (about half the weight of a grain of table salt)/mL in PBS; Solarbio, China) was added and incubated for 4 hrs. The MTT solution was then discarded, and formazan crystals were completely dissolved with 100 µL of 100% DMSO (Finar Chemicals, India). Absorbance readings were taken at 570 nm using a microplate reader (Thermo Fisher Scientific, USA). All experiments were performed with three technical replicates. Cell viability (%) was determined using the formula:

Cell viability (%) = [Absorbance of treated cells / Absorbance of untreated cells] × 100

Concentration and cell viability values were then used as input in the calculation of the dose–response curve and 50% cytotoxic concentration (CC _50_) using GraphPad Prism v10. Compounds exhibiting CC _50_ > 100 µM were considered non-cytotoxic and selected for further evaluation.

### *In silico* analyses

***Molecular docking.*** Molecular docking was conducted using the Maestro graphical user interface of the Schrsodinger Suite software package (Schrsödinger, LLC, NY, 2020-2). The suite offers several tools for protein preparation, active site identification, grid generation, ligand preparation, and molecular docking ( Moussa *et al.*, 2021).

***Ligand preparation.*** The 2-D structures of the compounds to be evaluated in SDF format were retrieved from PubChem and prepared for docking using LigPrep, in which the Epik Classic module was used to generate tautomers and possible ionization states at the default pH of 7.00 (+2.00), utilizing the OPLS4 force field ( Moussa *et al.*, 2021).

***Protein preparation.*** It has been established that BDQ is extruded by *Mtb* MmpS5-MmpL5 EP while CFZ and DOX are extruded by Rv1258c and MmpS5-MmpL5 EPs ( Laws *et al.*, 2022). While the MmpS5-MmpL5 EP is also present in *Msm*, the Rv1258c EP is absent in *Msm*. Nonetheless, MSMEG_5187 is a homologue of the *Mtb* Rv1258c gene expressed in *Msm* and effluxes tetracyclines ( Varadi *et al.*, 2024) ( Bowman & Ghosh, 2014; Li *et al.*, 2004). Homology models of *Msm* MmpL5 and MSMEG_5187 EPs were obtained from the AlphaFold DB ( Varadi *et al.*, 2024). Docking was carried out using MmpL5, rather than the entire MmpS5–MmpL5 complex, because MmpL5 constitutes the functional transmembrane transporter responsible for substrate extrusion. In contrast, MmpS5 is a small accessory protein that primarily stabilizes and regulates the activity of MmpL5 but does not form the drug translocation channel ( Ma *et al.*, 2020). Furthermore, accessory proteins such as MmpS5 are often structurally less well-characterized, and their absence does not substantially influence the binding pocket architecture of the transporter. For these reasons, focusing on MmpL5 provided the most biologically relevant and structurally reliable target for docking studies. They were prepared using the Protein Preparation tool to fix any issues with the structures ( Nada *et al.*, 2024). While assigning all bond orders using the Conserved Domains Database (CDD), disulfide bonds and zero-bond orders to metals were created. Additionally, missing loops and side chains were inferred using the Prime module, while the Epik module was used to generate hetero states. Before structure minimization using the OPLS4 force field, hydrogen bond assignments were optimized using PROPKA. Finally, water molecules within a 5Å radius of heteroatoms were deleted.

***Active site prediction and grid generation.*** The Sitemap wizard was used to predict likely binding sites in the homology models with sites with scores of a minimum of 15 site points presumed to be probable binding sites ( Ge *et al.*, 2024; Nath *et al.*, 2022). Thereafter, for docking, 3-D grids of potential binding sites with a druggability score of at least 0.7 were generated using Glide’s Receptor Grid Generation tool.

***Ligand docking.*** The prepared ligands were docked onto *Msm* MmpL5 and MSMEG_5187 3-D models using the Glide Ligand Docking tool ( Shamsian *et al.*, 2024). To scale the van der Waals radii, the default partial charge cut-off and scaling factor of 0.15 and 0.20, respectively, were used. Ligand sampling of the binding site was set to be flexible. For the ranking of each ligand’s docking pose, the Extra Precision (XP) docking score function, which included Epik state penalties, was used ( Lokwani *et al.*, 2020)

### Molecular Dynamics (MD) simulations

The MD simulations of the *Msm* MmpL5 and MSMEG_5187 apoproteins and their top-ranked docked complexes with the potential EIs were performed using Desmond, the GPU-powered high-performance MD engine, for 100 ns ( Bharadwaj *et al.*, 2024a). *Msm* has widely been used as a robust model in screening studies involving *Mycobactera* species such as *Mycobacterium leprae*, *Mtb* and *Mycobacterium bovis*. It is a directly budding, eco-friendly organism and does not cause human infection ( Joseph Antony Sundarsingh *et al.*, 2020). Given that MmpL5 and MSMEG_5187 are transmembrane proteins, a dipalmitoylphosphatidylcholine (DPPC; 350K) membrane was built around their helixes using the System Builder wizard that additionally built an orthorhombic-shaped solvent system with a TIP3P water model. The solvent system’s energy was minimized using the OPLS4 force field. Sodium and chloride ions were added to neutralize the system before the addition of 0.15M salt to mimic the pH of the intracellular environment ( Moussa *et al.*, 2021). The system was equilibrated using the isothermal-isobaric (NPT) ensemble to 1.01 bar and 300K and subjected to MD simulations for 100 ns. To determine the conformational stability of the ligand-protein complex and key ligand-protein contacts responsible for the potential EI activity observed *in vitro*, a simulation interaction analysis was performed.

### Molecular Mechanics Generalized Born Surface Area (MM-GBSA) calculations

The MM-GBSA method was utilized to calculate the binding free energy of the protein-ligand complexes to determine if they were conformationally stable throughout the MD simulations and predict the ligand-binding affinities ( Genheden & Ryde, 2015). The thermal mmgbsa.py script of the Prime Module used to perform the MM-GBSA calculation ( Bharadwaj *et al.*, 2024b). Briefly, frames of the protein-ligand complexes for the last and first 10 ns were extracted from the MD trajectory and the individual energy modules of the protein, ligand, and the complex computed ( Bharadwaj *et al.*, 2024b). The total MM-GBSA binding free energy was computed using the formula below:


$$\text{Δ}G_{bind}=G_{complex}-(G_{protien}+G_{ligand})$$

Where,

Δ *G*_bind_: binding free energy; G _complex_: complex’s free energy; G _protein_: target protein’s free energy; and, G _ligand_: ligand’s free energy.

## Results and discussion

### Potential EIs identified and their predicted physicochemical properties

**Figure 1.**
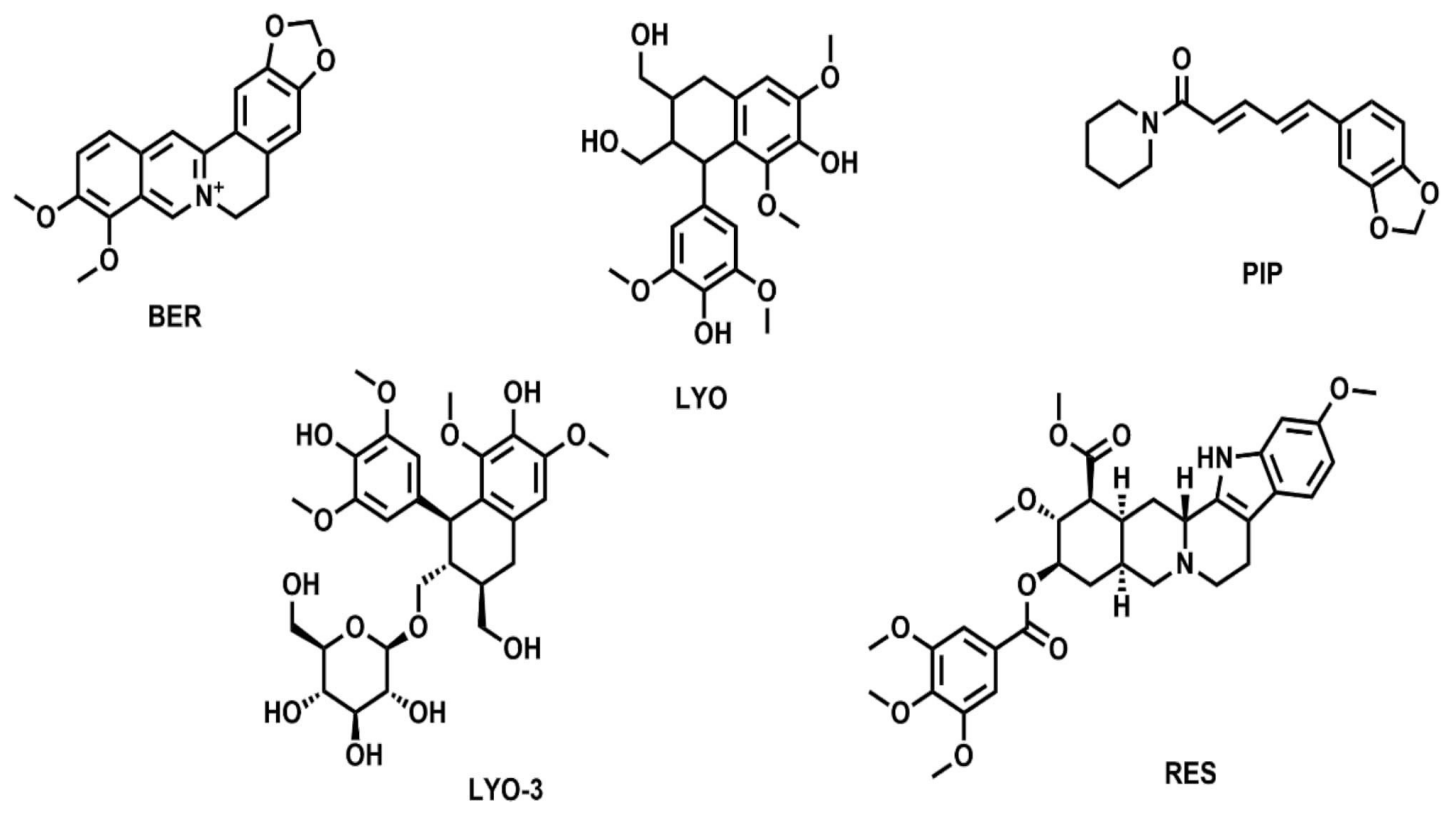
Chemical structures of the five compounds screened for antimicrobial activity, namely berberine (BER), piperine (PIP), reserpine (RES), lyoniresinol 3-Alpha-O-Beta-D-glucopyranoside (+)- (LYO-3) and lyoniresinol (LYO).

Collectively, 89 NP-based compounds, including known EIs such as RES, BER and PIP were identified from published literature and compound databases. Of the 89 compounds, RES, BER, PIP, LYO, and LYO-3 were readily available for antimicrobial screening. Their chemical structures and predicted physicochemical properties are shown in Figure 1 and Table 1, respectively.

**Table 1.** Predicted physicochemical properties of the five compounds tested for antimicrobial activity.

Compound ID	MSMEG_5187 docking score (kcal/mol)	MmpL5 docking score (kcal/mol)	M _W_	LogP	HIA category	HBD	HBA	TPSA
LYO-3	-7.6	>-7.0	582.6	-0.5	-	7	13	197
LYO	>-7.0	-7.5	420.5	1.5	-	4	8	117.8
PIP	-6.4	-5.3	285.3	2.6	+	0	4	38.8
BER	-5.6	-7.1	336.4	3.6	-	0	5	40.8
RES	-5.4	-5.7	608.7	3.4	+	1	11	117.8

Molecular weight (MW) <500; LogP <5; hydrogen bonding donors (HBD) <5; hydrogen bonding acceptors (HBA) <10; Topological polar surface area (TPSA) 26-225); HIA category: predicted human intestinal absorption, + indicates absorption ≥ 30%; - indicates absorption < 30%; violations of Lipinski’s Rule of Five are highlighted with a red shading.

Prediction of physicochemical properties revealed that LYO, PIP, and BER conform to Lipinski’s Rule of Five (Ro5) ( Lipinski, 2004). Two and three violations of this rule were observed for RES and LYO-3 respectively. Two or more violations of Lipinski’s Ro5 by synthetic compounds points to probable poor oral bioavailability which often leads to the de-prioritization of such compounds if intended for oral administration ( Benet, 2023). However, the rule does not apply to NPs because of their evolution-guided bioactivity and the use of membrane transporters for cell entry ( Beutler, 2019). As such, the five compounds were evaluated for their ability to potentiate the antimycobacterial activity BDQ, CFZ and DOX using *Msm* to infer their potential EI activity.

### Combination assays of the potential EIs with standard anti-TB drugs

Synergy was observed when BDQ, CFZ and DOX were combined with the putative EIs against *Msm*, as demonstrated on Table 2. The MIC _99_ of BDQ, CFZ and DOX were 0.433µg/mL, 0.039µg/mL, and 16µg/mL, respectively when tested singly. When combined with BER, LYO, and LYO-3, BDQ’s MIC _99_ was lowered by 8-, 64- and 32-fold with FICI values of 0.23, 0.18 and 0.3 respectively, indicating synergism between the EIs and BDQ. RES and PIP reduced the MIC _99_ of CFZ by 4 and 8-fold, resulting in a FICI value of 0.37 for PIP, pointing to synergism between PIP and CFZ. Furthermore, BER, RES, PIP, LYO and LYO-3 dropped the MIC _99_ value of DOX by 16-, 32-, 16-,16- and 16- fold (FICI = 0.31, N/A, 0.21, 0.19, 0.21) respectively. These results revealed that BER, LYO, LYO-3, PIP, and RES significantly enhanced the antimycobacterial activity of BDQ, CFZ, and DOX against *Msm*, putatively by disrupting efflux. Notably, the non-pathogenic *Mtb* surrogate, *Msm*, utilised in the assays shares numerous signalling and biosynthetic pathways with *Mtb* ( Joseph Antony Sundarsingh *et al.*, 2020), it is plausible that BER, PIP, RES, LYO, and LYO-3 may augment the antimycobacterial activity of BDQ, CFZ, and DOX in *Mtb*.

**Table 2.** Checkerboard combination assay results for BDQ, CFZ and DOX together with five known and potential EIs against *Msm*.

Drugs/compound	MIC _99_ (µg/ml) singly	MIC _99_ (µg/ml) combination	MIC _99_ (µg/ml) fold reduction of anti-TB drug	FIC	FICI
BDQ BER	0.217	0.022	**8**	0.102	**0.23**
	74.324	9.291		0.125	
BDQ RES	0.433	0.217	2	0.5	N/A
	>1363	>1363		N/A	
BDQ PIP	0.433	0.433	NC	1	2
	34.241	34.241		1	
BDQ LYO	0.433	0.011	**64**	0.026	**0.18**
	84.092	10.512		0.125	
BDQ LYO-3	0.433	0.022	**32**	0.05	**0.3**
	58.259	14.565		0.25	
CFZ BER	0.039	0.039	NC	1	2
	37.162	18.581		1	
CFZ RES	0.039	0.010	**4**	0.25	N/A
	>1363	>1363		N/A	
CFZ PIP	0.039	0.005	**8**	0.12	**0.37**
	34.241	8.560		0.25	
CFZ LYO	0.039	0.039	NC	1	2
	84.092	84.092		1	
CFZ LYO-3	0.039	0.039	NC	1	2
	58.259	58.259		1	
DOX BER	16	1	**16**	0.063	**0.31**
	37.162	9.291		0.25	
DOX RES	16	0.5	**32**	0.031	N/A
	>1363	>1363		N/A	
DOX PIP	16	1	**16**	0.063	**0.21**
	34.241	8.560		0.25	
DOX LYO	16	1	**16**	0.063	**0.19**
	84.092	10.512		0.125	
DOX LYO-3	16	1	**16**	0.063	**0.21**
	58.259	14.565		0.25	

In line with our results, several studies have demonstrated the utility of BER, PIP, and RES as potentiators of various antibacterial agnates against drug-resistant bacteria. For example, ( Li & Ge, 2023b) demonstrated that BER inhibits *Escherichia coli’s* MdfA, a major facilitator superfamily (MFS) EP, and elevates the intracellular concentration of ciprofloxacin. Moreover, it has been established that BER inhibits *Pseudomonas aeruginosa’s* MexXY multidrug efflux system and amplifies the antibacterial activity of aminoglycosides against multi-drug resistant strains ( Morita *et al.*, 2016). To the best of our knowledge, no studies have reported the potential mycobacterial efflux inhibition activity of BER. Consistent with this study’s findings, the probable efflux inhibition activity of PIP against *Msm* has been previously reported ( Jin *et al.*, 2011). Besides, the potential of PIP to disrupt efflux in other bacteria, including meticillin-resistant *Staphylococcus aureus,* carbapenem-resistant *P. Aeruginosa* and *Mtb* has been reported ( Liu *et al.*, 2019; Shaheen *et al.*, 2019a; Sharma *et al.*, 2010). Several studies have also demonstrated the ability of RES to boost the activity of various antibacterial agents by disrupting diverse EPs including the acriflavine resistance protein B (AcrB) transporter of the resistance nodulation division family ( Shaheen *et al.*, 2019b), Tet(K) in methicillin-resistant *Staphyloccocus aureus* (MRSA) ( Gibbons & Udo, 2000), and EPs in ciprofloxacin-resistant *Mtb* ( Huang *et al.*, 2013). Given that no studies have been published on the potential of LYO and LYO-3 as EIs, this study illustrates that plant families and analogs of known EIs may be utilized suitably as tractable starting points in the uncovering of novel EIs ( Seukep *et al.*, 2020).

### Cytotoxicity evaluation on vero E6 cells

**Figure 2.**
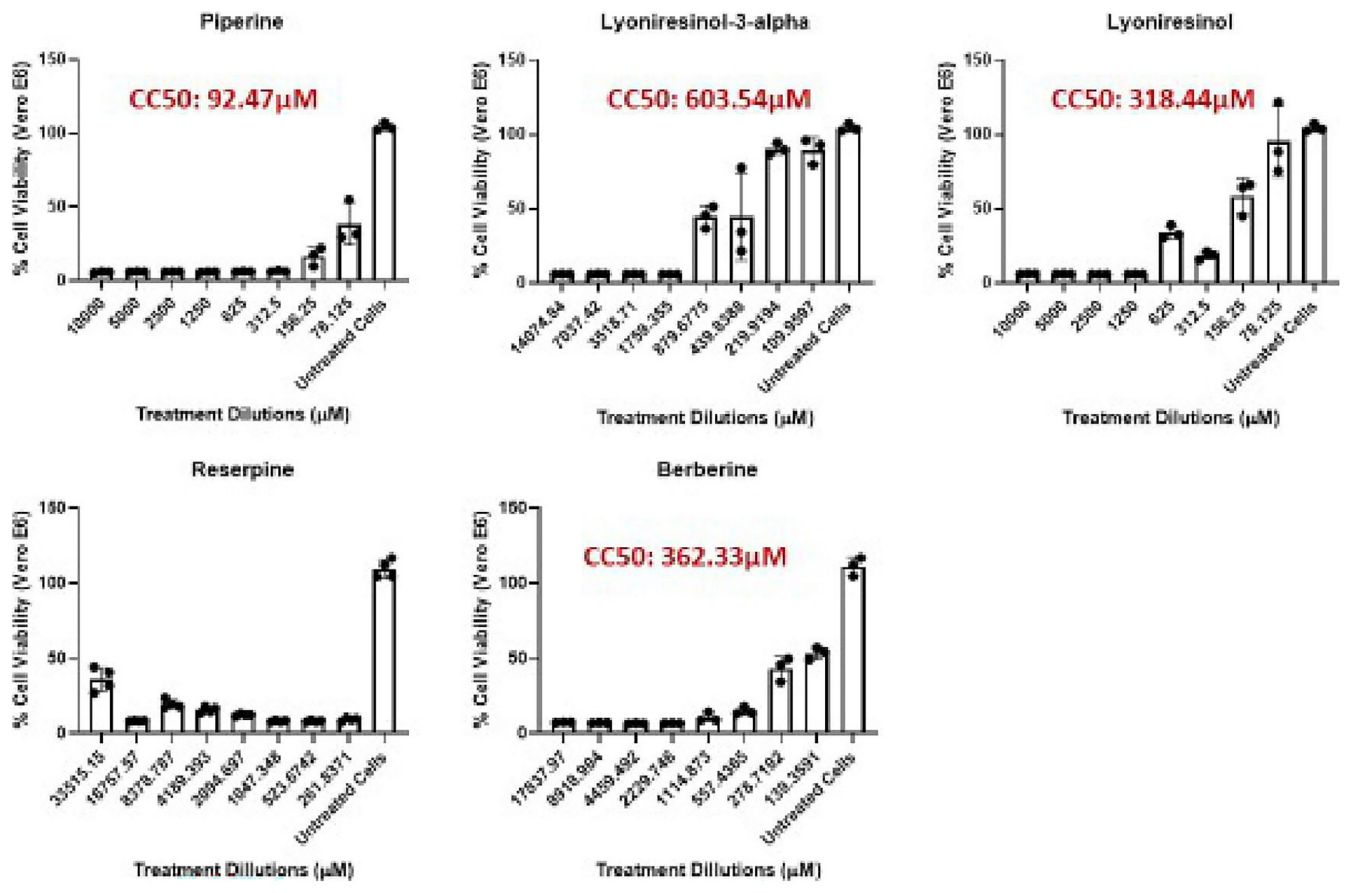
Cytotoxicity activity of potential and known EIs against Vero cells. The dose–response curves of the percentage Vero E6 cells viability following 48 hrs exposure to increasing concentrations of efflux inhibitors are shown. Data are presented as mean ± standard deviation of triplicate determinations. CC _50_ values were determined using nonlinear regression.

The cytotoxicity profiles of the five EIs were assessed using Vero E6 cells, and CC _50_ values were derived from dose-response curves ( Figure 2). Significant differences were observed among the compounds. PIP showed the lowest CC _50_ (92.47 µM), indicating higher cytotoxicity compared to the others. Cell viability dropped sharply with increasing concentrations, suggesting a narrow safety margin. This value is below the generally accepted non-toxic threshold of 100 µM in early drug discovery screens ( Shafiee *et al.*, 2021), implying that PIP would need highly potent antimycobacterial activity (i.e., low MIC) to achieve an acceptable selectivity index (SI). In contrast, LYO-3 demonstrated the best safety profile, with a CC _50_ of 603.54 µM. Cell viability stayed above 50% even at higher concentrations, indicating low cytotoxicity. Similarly, Lyoniresinol (CC _50_ = 318.44 µM) and BER (CC _50_ = 362.33 µM) showed moderate cytotoxicity, with CC _50_ values well above the 100 µM threshold, suggesting they are relatively safe for further testing. RES, however, exhibited strong cytotoxicity at low micromolar concentrations, and no reliable CC _50_ could be determined within the tested range.

### Molecular docking of potential EIs to infer the mechanism of action

Molecular docking, which predicts a ligand’s binding affinity to its target, is a widely used computational method for inferring the possible mode of action of potential drugs ( Agu *et al.*, 2023). In this study, BER, PIP, RES, LYO and LYO-3 were docked onto *Msm* MSMEG_5187 and MmpL5 EPs. It has been established that Mtb MmpL5 EP extrudes BDQ while CFZ and DOX are extruded by Rv1258c and MmpS5-MmpL5 EPs ( Laws *et al.*, 2022). While MmpS5-MmpL5 is also present in *Msm*, Rv1258c is absent in *Msm*. Nonetheless, MSMEG_5187 is a homologue of the Rv1258c EP expressed in *Msm* ( Li *et al.*, 2004). The *Msm* Mmpl5 and MSMEG_5187, modelled using SWISS-MODEL ( Waterhouse *et al.*, 2018), were docked with BER, PIP, RES, LYO, and LYO-3 to infer whether their observed synergistic activity with DOX, CFZ and BDQ in *Msm* could be attributed to their direct inhibition of these EPs.

### Homology models of Msm MSMEG_5187 and MmpL5

**Figure 3.**
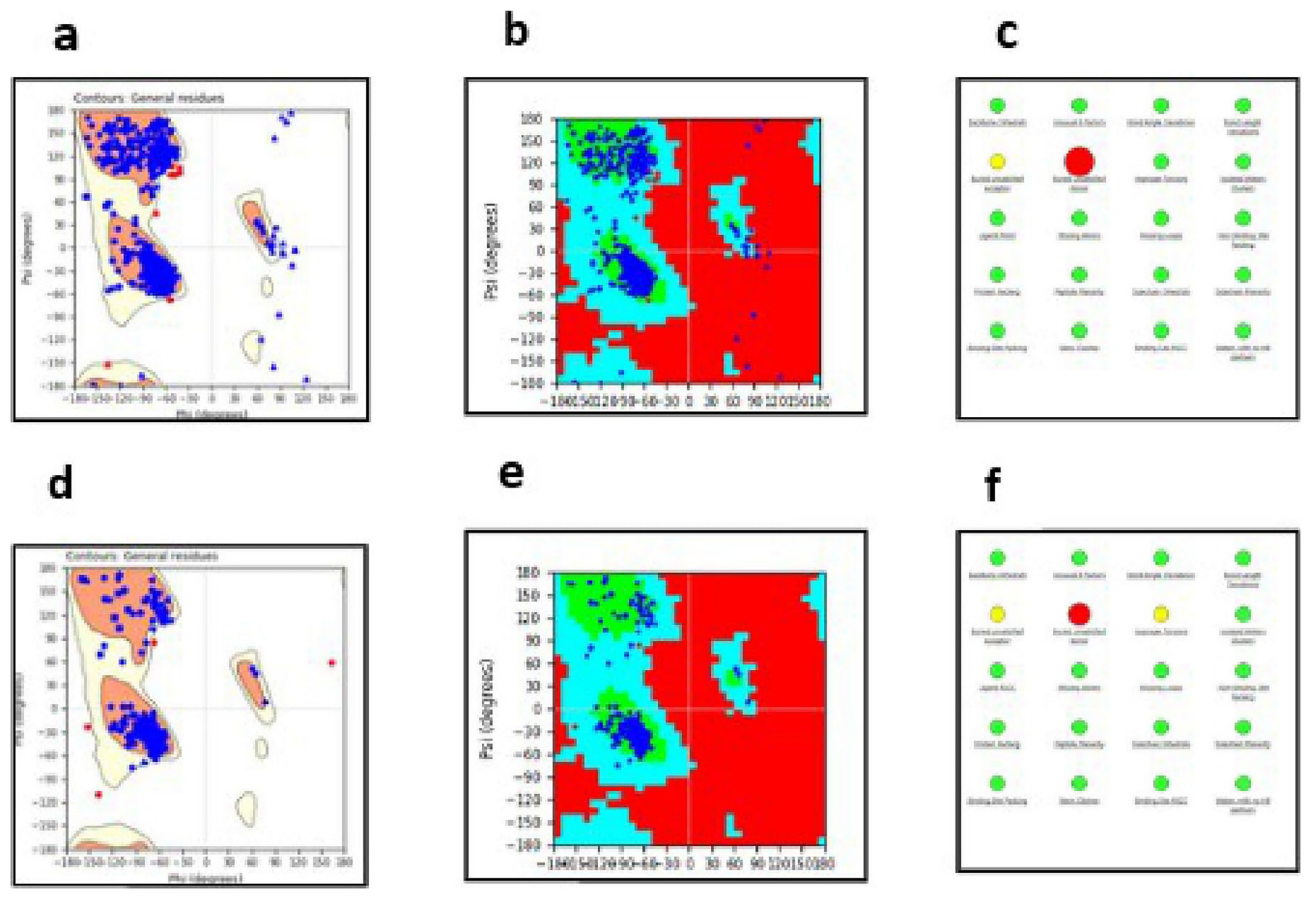
The Ramachandran plots of *Msm* MmpL5 model’s structural robustness are depicted in panels **a** and **b**, while the protein reliability report is shown in panel **c**. Msm MSMEG_5187 model’s Ramachandran plots are represented in panels **d** and **e** with its protein reliability report shown in panel **f**.

The quality of the *Msm* MSMEG_5187 and MmpL5 3-D models obtained using SWISS-MODEL was evaluated using the Protein Reliability tool in Maestro which assesses the quality and stability of a protein structure and outputs a Ramachandran plot and protein reliability report that highlight any modelling issues. Specifically, Ramachandran plots indicate a protein structure or model stereochemical quality by highlighting whether the amino acid residues are in disallowed, allowed, or favoured regions of the structure ( Hollingsworth & Karplus, 2010). The Ramachandran plots and protein reliability reports for *Msm* MmpL5 and MSMEG_5187 are illustrated in Figure 3.

The models obtained are structurally robust given that >98% of their amino acid residues fell within the allowed and favoured regions. Also, the models only exhibit minor structural issues mainly in buried unsatisfied donors and acceptors. None of these structural violations was localized in the predicted active sites. Thus, the two models were appropriate for ligand docking.

### Active site prediction and ligand docking

Prediction of the potential ligand binding sites in MmpL5 and MSMEG_5187 was performed using SiteMap. Of the five binding sites predicted in *Msm* MmpL5, three (Sites 1, 2, and 3) had a druggability score and site score above 0.7. Similarly, all three predicted sites in Rv1258c (MSMEG_5187) also demonstrated druggability and site scores above 0.7. Although *Msm* is a non-pathogenic surrogate, its EP homologs are widely used in structural and functional studies because of their high sequence and structural conservation with *Mtb* EPs ( Hartkoorn *et al.*, 2014). To help identify the most probable binding site in both systems, verapamil (VER), a known inhibitor of these EP, was docked as a reference ligand ( Laws *et al.*, 2022). This analysis revealed Site 1 as the most probable binding site for inhibitor interaction. The MIC _99_ fold reduction, FICI in relation to the binding affinities for each evaluated compound, is depicted in Table 3.

**Table 3.** MIC _99_ fold reduction and FICI values for BDQ, CFZ and DOX in combination with potential efflux inhibitors (EIs) alongside molecular docking scores for the compounds against *Msm* MmpL5 and MSMEG_5187.

Potential EI	MIC _99_ (µg/ml) fold reduction and FICI values of BDQ, CFZ, and DOX in combination with potential EIs						*EI Docking Scores (kcal/mol)	
	BDQ		CFZ		DOX		MmpL5	Rv1258c
	Fold reduction	FICI	Fold reduction	FICI	Fold reduction	FICI		
BER	8	0.23	NR	N/A	16	0.31	-3.64	-4.36
RES	2	N/A	4	N/A	32	N/A	-4.40	-2.24
PIP	NR	N/A	8	0.37	16	0.21	-4.09	-6.57
LYO	64	0.18	NR	N/A	16	0.19	-8.24	-4.62
LYO-3	32	0.3	NR	N/A	16	0.21	-6.97	-9.22

The docking results suggested that LYO and LYO-3 are probable inhibitors of MmpL5, based on their docking scores. These results putatively support the *in vitro* results that revealed LYO+BDQ and LYO-3+BDQ combinations as synergistic with FICI values of 0.18 and 0.3, respectively. The docking scores for RES, PIP and BER were greater than the score for VER, implying weak binding onto MmpL5. In line with these results, no synergy was observed for the RES+BDQ and PIP+BDQ combinations. The docking score obtained for BER indicates that this compound is unlikely to inhibit MmpL5 and, as such, the synergism observed *in vitro* with BDQ could be due to the disruption of another EP or interference with the electron transport chain ( Sharma *et al.*, 2010).

Results obtained for the docking of PIP onto MSMEG_5187 indicate that this compound probably inhibits this EP, which correlates with *in vitro* results that showed PIP to enhance the antimycobacterial activity of CFZ but not BDQ. Several studies have established PIP as an inhibitor of the *Mtb* Rv1258c ( Cloete *et al.*, 2018; Laws *et al.*, 2022; Sharma *et al.*, 2010), which further supports the docking results on the MSMEG_5187 homolog. While the docking results imply that LYO-3 could be a stronger inhibitor of MSMEG_5187 than PIP, there was no observed change in the MIC _99 _of CFZ when used together with LYO-3. The docking results of BER, RES, and LYO point to weak affinity for MSMEG_5187, which supports the *in vitro* results that showed no synergism with CFZ. Remarkably, synergism was observed when each of the compounds was combined with DOX, which is also extruded by Rv1258c, implying that the compounds could be inhibiting the efflux function of Rv1258c.

### Molecular Dynamics (MD) simulations

Unlike molecular docking, molecular dynamics (MD) simulations capture the flexible and dynamic nature of receptor protein and solvent effects on ligand-protein contacts due to the solvation of the protein, which increases the accuracy of the predicted ligand binding affinity and binding poses ( Guterres & Im, 2020). As such, MD simulations provide finer information about key protein-ligand interactions. To better understand the key interactions of the potential EIs with the EPs, and the conformational stability of the resulting complexes, MD simulations of each potential EI in complex with either MmpL5 or MSMEG_5187 were conducted using Desmond for 100 ns ( Moussa *et al.*, 2021).

### MmpL5

**Figure 4.**
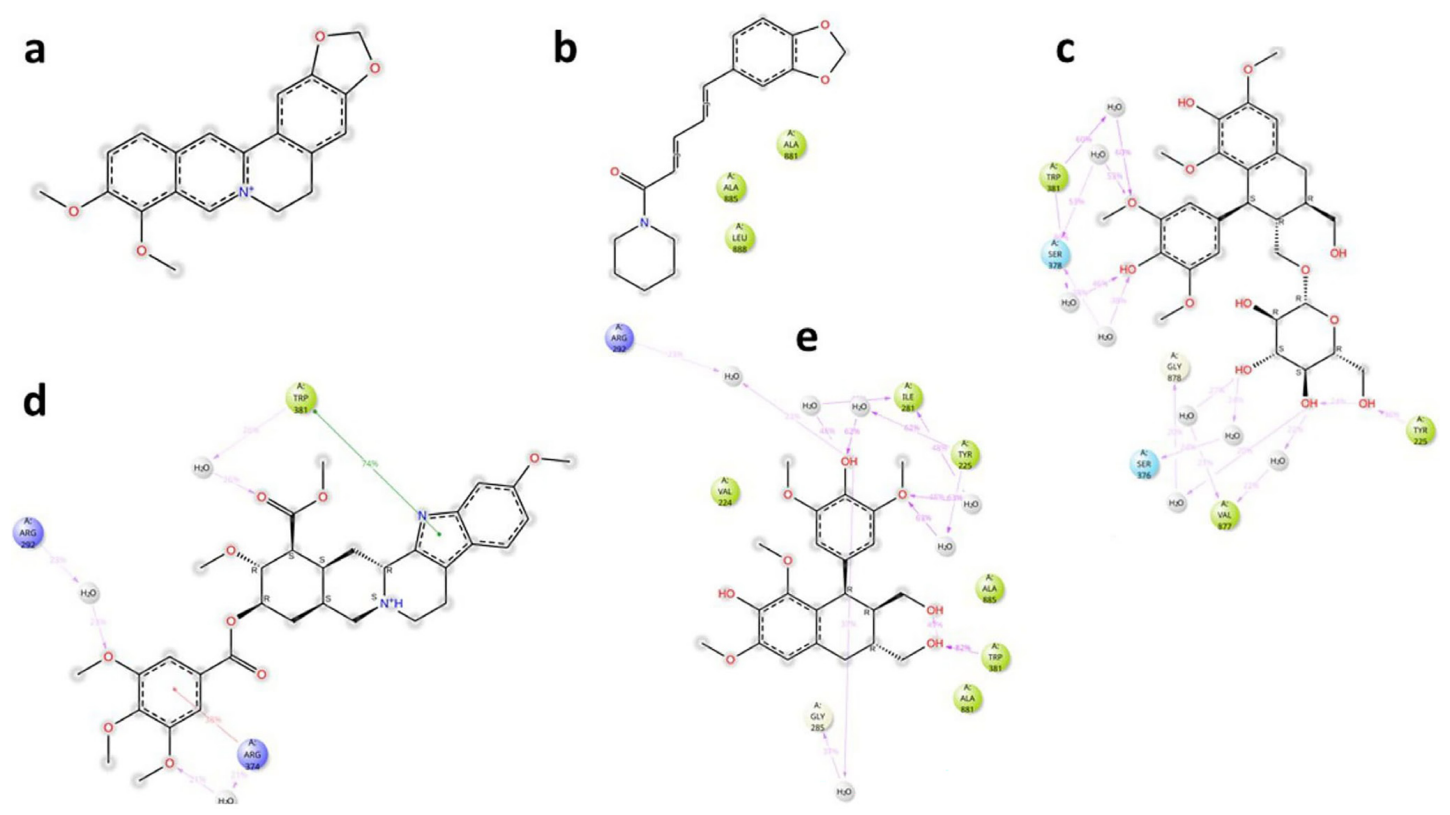
Key ligand-protein interactions for *Msm* MmpL5 in complex with BER ( **a**), PIP ( **b**), LYO-3 ( **c**), RES ( **d**), and LYO ( **e**).

The key interactions of the potential and known EIs with MmpL5 amino acid residues, together with their time duration (% of total simulation time) are shown in Figure 4.

Analysis of the MmpL5-BER complex revealed that no key interactions were formed with any of the binding site’s residues that lasted for at least 20 ns, which means that even very transient interactions of BER with MmpL5 may be adequate to cause functional disruption, as evidenced by the synergism observed between BDQ and BER in the combination assays. The other putative EIs displayed interactions with some key residues in the MmpL5 binding site that lasted for at least 20 ns as depicted in Table 4. Although PIP formed three hydrophobic contacts, these interactions seem to be insufficient to inhibit MmpL5 as PIP was not synergistic with BDQ in the combination assays. LYO, LYO-3 and RES formed interactions with several MmpL5 amino acid residues for over 20% of the simulation time which indicates that these compounds are likely inhibitors of *Msm* MmpL5 and plausibly explains their ability to lower the MIC _99_ of BDQ in the combination assays. 

**Table 4.** Summary of key ligand-protein interactions for the *Msm* MmpL5 in complex with BER, PIP, LYO-3, RES and LYO.

Compound	H bonds/salt bridges			Hydrophobic interactions		
	No.	Type	Amino acid residues involved	No.	Type	Amino acid residues involved
BER	0	-	-	0	-	-
PIP	0	-	-	3	Alkyl	ALA881, ALA885, ALA888
RES	3	Water-mediated H bond	ARG292, ARG374, TRP381	1	Pi-Pi stacking	TRP 381
LYO	4	Water-mediated H bond	TYR225, ILE281, GLY285, ARG292	6	Alkyl	VAL224, TYR225, ILE 281, TRP381, ALA881, ALA885
		H bond	TRP381			
LYO-3	6	Water-mediated H bond	TYR225, SER376, SER378, TRP381, VAL877, GLY878	3	Alkyl	TRY225, TRP381, VAL877

**Figure 5.**
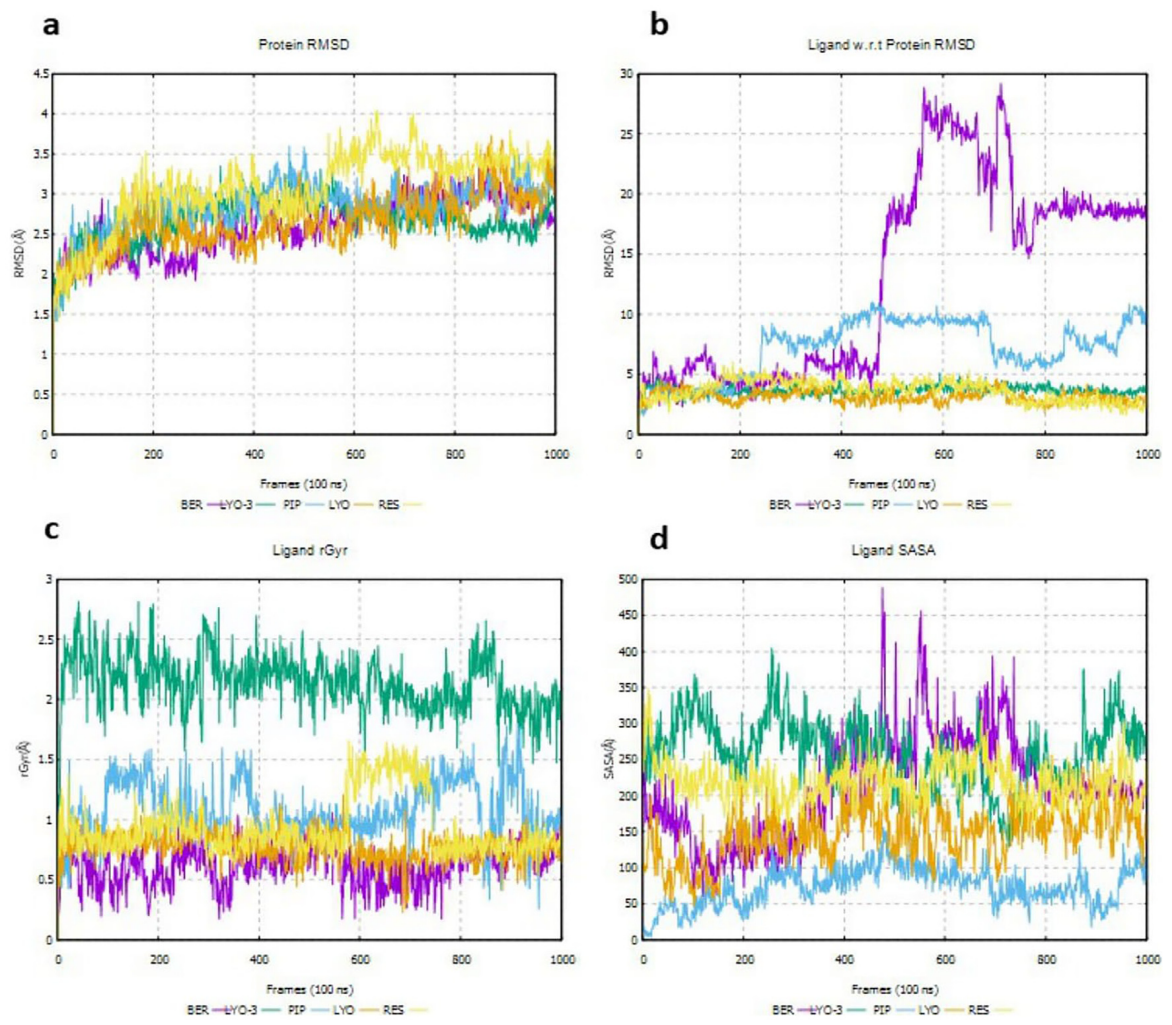
Deviations in protein RMSD for EI-MmpL5 complexes ( **a**), protein-ligand RMSD for EI-MmpL5 complexes ( **b**), rGyr for the EIs ( **c**) and SASA for the EIs ( **d**) throughout the molecular dynamics simulation.

The formation of ligand interactions with key amino acid residues in a protein’s binding cannot be solely used to infer disruption of protein function. Hence, the Root Mean Square Deviation (RMSD) of the protein and the ligand with respect to (w.r.t) the protein, as well as the radius of gyration (rGyr) and solvent accessible surface area (SASA) of the ligand, were evaluated to determine the conformational stability of the EI and EI-MmpL5 complexes throughout the simulation. The results are shown in Figure 5.

The protein RMSD of a complex assesses the structural conformation variation of the protein during the simulation and equilibration ( Manandhar *et al.*, 2022). As shown in Figure 5A, the protein RMSDs for the BER, PIP, LYO, and LYO-3 complexes were ≤ 3 Å and stabilized around a fixed value indicating that the system was equilibrated and the protein did not undergo a huge conformational change due to ligand binding. The RMSD for RES complex was approximately 3–4 Å, which is allowed given the size of MmpL5. 

The ligand RMSD w.r.t the protein’s binding pocket measures the RMSD of its heavy atoms after aligning the ligand-protein complex to the reference’s protein backbone, indicating its stability inside the binding site ( Nada *et al.*, 2024). As shown in Figure 5B, the ligand RMSD w.r.t the protein’s binding pocket for LYO, LYO-3, and RES was estimated at 5 Å throughout the simulation. The fact that the ligand RMSDs are not significantly greater than the protein RMSD indicates that the compounds are stable in the MmpL5 binding site. In contrast, the ligand RMSD of PIP w.r.t protein was about 5 Å in the initial 200 frames but increased thereafter to approximately 10 Å indicating that PIP most likely diffused away from its initial binding site after 200 frames ( Manandhar *et al.*, 2022). The lack of stability of PIP in MmpL5 binding site may explain the lack of synergism with BDQ in the combination assays. Similarly, the high BER RMSD value after about 450 frames (10 – 30 Å), indicates that BER might have diffused away from its original binding site ( Allegra *et al.*, 2021). This contradicts the synergism observed between BDQ and BER in the combination assays.

The rGyr of a ligand indicates the average spread of its atoms from its centre of mass, indicating its compactness and flexibility inside the protein’s binding site. As shown in Figure 5C, all the compounds displayed a low rGyr (<3 Å,) indicating tight packing inside the binding site. A ligand’s SASA measures the surface area that is exposed to the solvent during an MD simulation ( Wang & Hou, 2012). A large change in SASA might indicate that the conformation of the protein’s binding site changes upon ligand binding. Only BER exhibited a vast change in SASA values during the simulation (200 – 500 Å), suggesting that MmpL5 undergoes an enormous conformation change upon BER binding. Studies have established that the conformational alteration that occurs in many proteins upon ligand binding may jettison their normal functions ( Marsh & Teichmann, 2011). Thus, it is probable that even though BER lacks key interactions with MmpL5 binding site residues for at least 20% of the simulation time, its transient binding triggers conformational changes that hamper the proper functioning of MmpL5. This may explain the synergism observed when BER was combined with BDQ.

**Figure 6.**
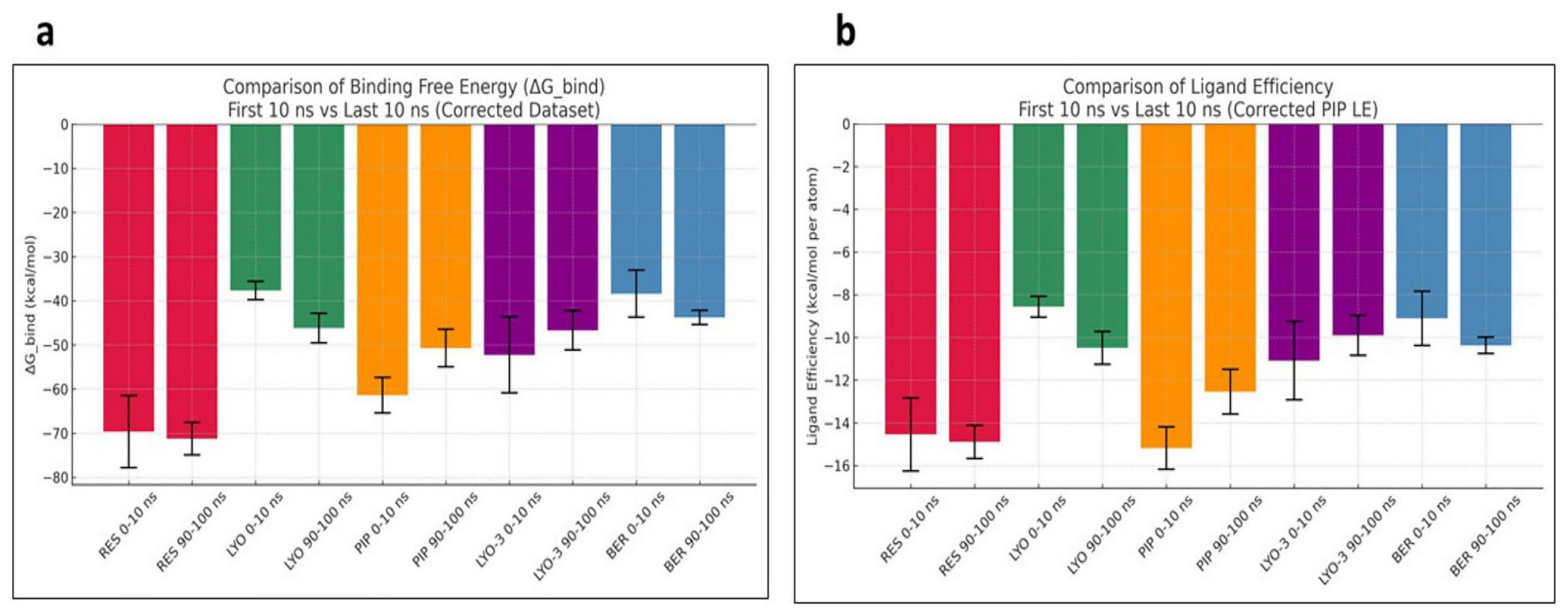
Comparison of MM-GBSA binding free energy ( **a**) and ligand efficiency ( **b**) for the *Msm* MmpL5 bound to BER, RES, PIP, LYO-3 and LYO in the first and last 10 ns of the molecular dynamics simulation.

To further probe the conformational stability of the EI complexes, MM-GBSA calculations were performed to estimate the average binding free energies of their MD snapshots during the first and last 10 ns ( Genheden & Ryde, 2015). The results, as provided in Figure 6, show that the compounds had differential binding stabilities during the simulation. RES demonstrated strong binding throughout the simulation, with the ΔG_bind and ligand efficiency decreasing from –69.61 to –71.24 kcal/mol and –14.54 to –14.89 kcal/mol, respectively. These results indicated that the RES-Mmpl5 complex was conformationally stable, supporting the observed synergism in the in vitro assays. Similarly, the complex of Mmpl5 with BER and LYO appeared to be conformationally stable since both their ΔG_bind and ligand efficiency improved during the simulation. In contrast, Mmpl5-PIP and -LYO-3 complex became unstable as the simulation progressed, evidenced by their ΔG_bind and ligand efficiency becoming less negative. 

Decomposition of the MM-GBSA energetic terms, as shown in Table 5, suggested that unfavorable solvation energies for LYO, LYO-3, and PIP offset the stabilizing effect of the van der Waals and lipophilic interactions. These findings suggest that structural optimizations might be needed to overcome the solvation penalties ( Kraml *et al.*, 2019).

**Table 5.** Summary of MM-GBSA analysis for *Msm* MmpL5 in complex with BER, PIP, LYO-3, RES and LYO.

Complex	Time (ns)	ΔG bind	Coulomb	Covalent	H-bond	Lipo	Solv_GB	VdW
RES	0-10	-69.61 ± 8.19	63.34 ± 29.68	3.83 ± 2.42	-0.32 ± 0.29	-18.95 ± 1.48	-48.94 ± 30.43	-60.59 ± 5.87
	90-100	-71.24 ± 3.69	62.25 ± 37.12	3.36 ± 1.45	-0.43 ± 0.28	-19.00 ± 0.84	-49.49 ± 38.01	-59.19 ± 4.26
LYO	0-10	-37.67 ± 2.06	-9.74 ± 5.40	3.38 ± 2.47	-1.18 ± 0.47	-13.73 ± 1.03	21.46 ± 5.20	-37.49 ± 2.86
	90-100	-46.17 ± 3.37	-11.48 ± 10.25	3.49 ± 1.27	-1.15 ± 0.72	-16.51 ± 1.79	21.98 ± 7.66	-42.01 ± 3.12
PIP	0-10	-61.40 ± 4.02	-15.39 ± 2.89	1.25 ± 1.55	-0.64 ± 0.29	-22.05 ± 1.66	23.38 ± 1.93	-46.67 ± 1.87
	90-100	-50.70 ± 4.25	-4.76 ± 3.12	1.54 ± 0.84	-0.09 ± 0.16	-21.60 ± 1.39	17.44 ± 1.28	-41.75 ± 2.08
LYO-3	0-10	-52.24 ± 8.64	-23.78 ± 9.62	2.71 ± 2.37	-1.81 ± 0.88	-17.80 ± 3.08	29.97 ± 5.71	-41.52 ± 2.86
	90-100	-46.68 ± 4.45	-25.07 ± 8.22	2.27 ± 3.39	-1.55 ± 0.53	-13.10 ± 1.31	34.60 ± 7.33	-43.72 ± 3.94
BER	0-10	-38.38 ± 5.34	172.20 ± 14.42	2.31 ± 1.47	-0.12 ± 0.15	-11.60 ± 1.04	-16.38 ± 11.48	-35.34 ± 2.49
	90-100	-43.77 ± 1.58	38.21 ± 5.87	0	1.16 ± 0.68	-15.31 ± 0.49	-33.57 ± 6.88	-33.45 ± 1.27

ΔG bind – binding free energy, Coulomb - coulomb energy, Covalent - covalent binding energy, VdW - Van der Waals energy, Lipo - lipophilic energy, Solv_GB - Generalized Born electrostatic solvation energy, H-bond - hydrogen-bonding energy. All energies are in kcal/mol.

Collectively, the MD and MM-GBSA findings imply that RES, BER, and LYO are the most promising potential inhibitors of *Msm* Mmpl5. While studies have already established that RES and BER are EPIs ( Li & Ge, 2023a; Shaheen *et al.*, 2019a), the efflux inhibition potential of LYO has not been explored. Additionally, the targets of RES and BER in mycobacteria have not yet been elucidated. Therefore, additional enzymatic assays are warranted to confirm whether the compounds inhibit Mmpl5 as suggested by the computational results. 

### MSMEG_5187

**Figure 7.**
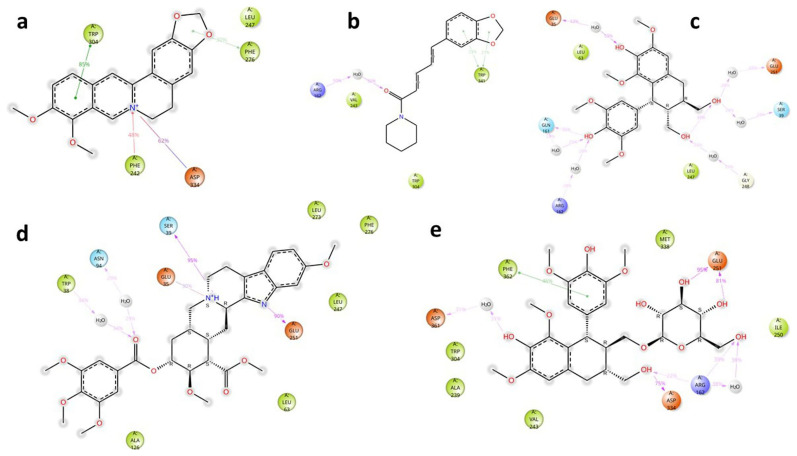
Key ligand-protein interactions for *Msm* MSMEG_5187 in complex with BER ( **a**), PIP ( **b**), LYO ( **c**), RES ( **d**) and LYO-3 ( **e**) for at least 20% of the simulation time.

The key interactions of the potential and known EIs with MSMEG_5187 amino acid residues, together with their time duration (% of total simulation time) are shown in Figure 7. Analysis of ligand-protein interactions for the MSMEG_5187 complexes indicated that all the ligands formed key interactions with several amino acid residues inside the protein’s binding site for at least 20 ns. The key interactions for each ligand are summarized in Table 6.

**Table 6.** Summary of key ligand-protein interactions for *Msm* MSMEG_5187 in complex with BER, PIP, LYO-3, RES and LYO.

Compound	H bonds/salt bridges			Hydrophobic interactions		
	No.	Type	Amino acid residues involved	No.	Type	Amino acid residues involved
BER	1	Salt bridge	PHE242	2	Pi-Pi stacking	TRP304, PHE276
				1	Pi-cation	PHE242
				4	alkyl	PHE242, LEU247, PHE276, TRP304
PIP	1	H bond	ARG162	1	Pi-Pi stacking	TRP341
				3	alkyl	VAL243, TRP341, TRP304
RES	2	Water-mediated H bond	ASN29, TRP38	6	alkyl	TRP38, LEU63, ALA126, LEU247, PHE276, LEU273
	2	H bond	SER39, GLU251			
	1	Salt bridge	GLU35			
LYO	6	Water-mediated H bond	GLU35, SER39, ARG162, GLN161, GLY248, GLU251	2	Alkyl	LEU63, LEU247
	1	H bond	GLN161			
LYO-3	2	Water-mediated H bond	ARG162, ASP361	1	Pi-Pi stacking	PHE362
	4	H bond	ARG163, GLU251, ASP334	6	Alkyl	VAL243, ILE250, ALA239, TRP304, MET338, PHE362

**Figure 8.**
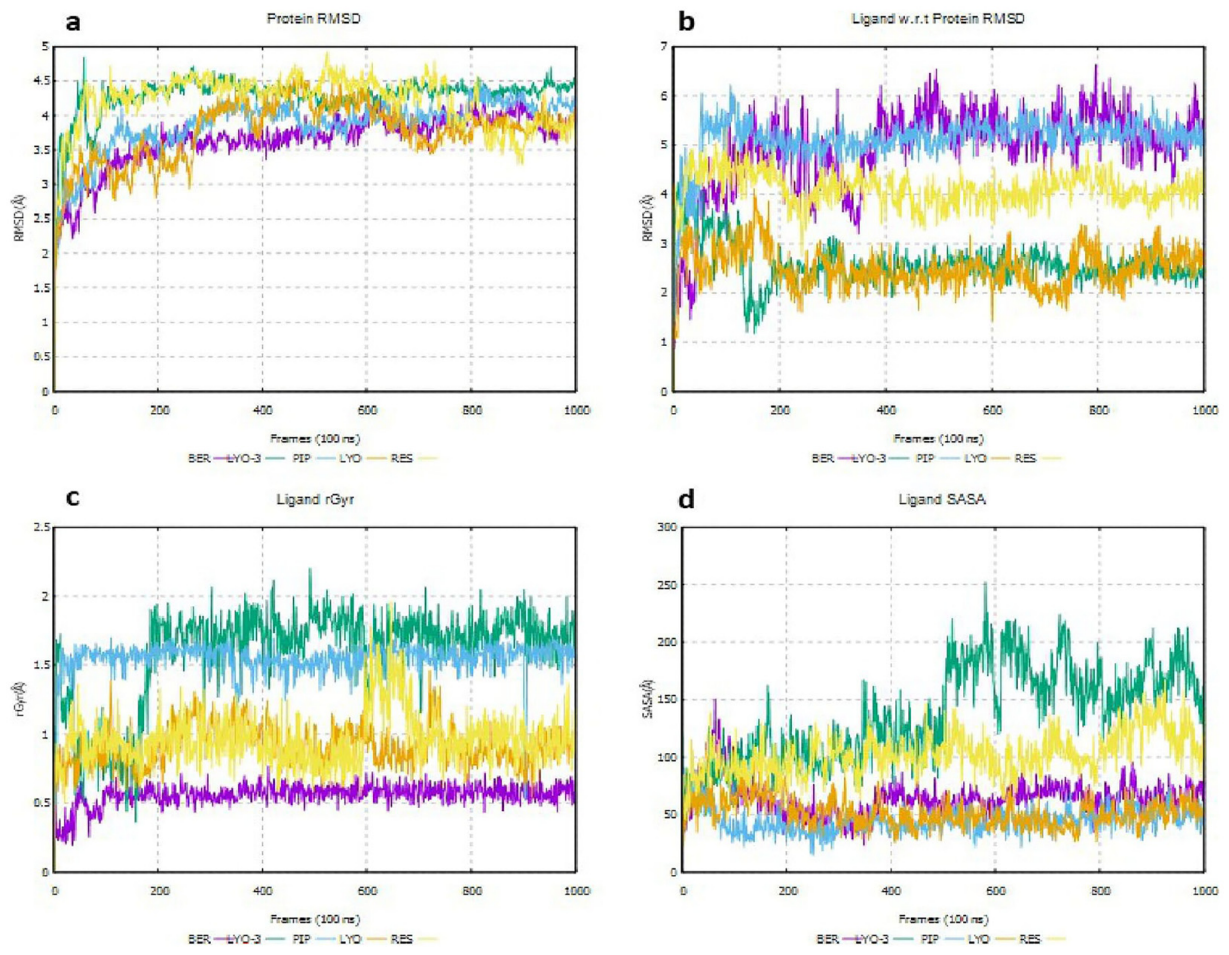
Deviations in protein RMSD for EI-MSMEG_5187 complexes ( **a**), protein-ligand RMSD for EI-MmpL5 complexes ( **b**), rGyr for the EIs ( **c**) and SASA for the EIs ( **d**) throughout the molecular dynamics simulation.

The RMSD of the protein and the ligand with respect to (w.r.t) the protein, as well as the rGyr and SASA of the ligand, were assessed to determine whether the EI and EI-MmpL5 complexes were conformationally stable throughout the simulation, as shown in Figure 8.

As illustrated in Figure 8B, the RMSD values obtained for MSMEG_5187 in complex with all the ligands indicated that the ligands were stable in the protein’s binding pocket given that the ligand RMSD minimally deviated from that of the protein ( Figure 8A) throughout the simulation ( Guterres & Im, 2020). The low rGyr ≤ 2 Å ( Figure 8C) obtained with all the compounds indicate tight packing within the binding site. LYO-3 exhibited a small increase (100 – 200 Å) in its SASA values during the simulation ( Figure 8D), which suggests that its binding onto MSMEG_5187 alters the protein’s conformation after 40 ns to more expose it to solvent.

**Figure 9.**
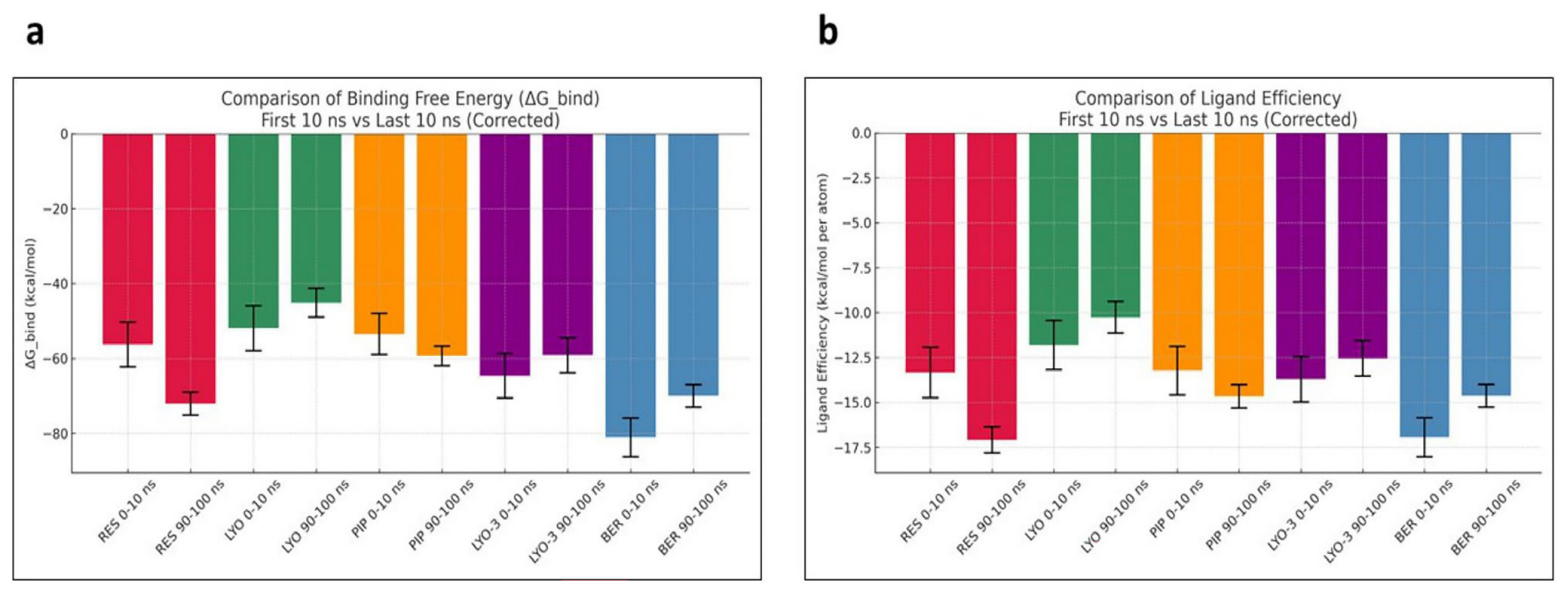
Comparison of MM-GBSA binding free energy ( **a**) and ligand efficiency ( **b**) for Msm MSMEG_5187 in complex with BER, RES, PIP, LYO-3 and LYO in the first and last 10 ns of the molecular dynamics simulation.

To further evaluate the conformational stability of the EI complexes, MM-GBSA calculations were performed to estimate the average binding free energies of their MD snapshots during the first and last 10 ns ( Genheden & Ryde, 2015). The results are provided in Table 7 and Figure 9. As shown in Figure 9, PIP formed a conformationally stable complex with MSMEG_5187, as the binding free energy (ΔG_bind) and ligand efficiency decreased during the simulation from -53.51 to -59.31 kcal/mol and 13.23 to -14.66 kcal/mol, respectively. Similarly, the ΔG_bind and ligand efficiency of RES decreased during the simulation from -56.26 to -72.06 kcal/mol and 13.33 to -17.08 kcal/mol, respectively. These results support the synergism observed in the *in vitro* assays when RES and PIP were used together with DOX and CFZ, known substrates of Rv1258c. In contrast, the ΔG_bind and ligand efficiency of LYO, LYO-3, and BER complexes increased during the simulation, suggesting they were not conformationally stable and explaining why no synergism was observed when they were combined with CFZ in the *in vitro* assays. Decomposition of the MM-GBSA energetic terms, as shown in Table 7, suggested that lipophilic and van der Waals stabilized the EI complexes, while the solvation penalties destabilized all the complexes except RES complex. 

**Table 7.** Summary of MM-GBSA analysis for *Msm* MSMEG_5187 in complex with BER, PIP, LYO-3, RES and LYO.

Complex	Time (ns)	ΔG bind	Coulomb	Covalent	H-bond	Lipo	Solv_GB	VdW
RES	0–10	-56.26 ± 5.96	42.79 ± 11.38	0.92 ± 0.93	-0.08 ± 0.16	-17.67 ± 1.47	-38.18 ± 10.19	-40.63 ± 2.08
	90–100	-72.06 ± 3.07	57.28 ± 18.21	0.39 ± 0.62	-0.01 ± 0.01	-23.18 ± 0.88	-51.48 ± 17.15	-47.99 ± 1.17
LYO	0–10	-51.93 ± 6.05	-17.93 ± 5.05	4.15 ± 1.07	-1.36 ± 0.63	-21.99 ± 2.45	36.98 ± 3.82	-51.13 ± 3.24
	90–100	-45.15 ± 3.83	-15.21 ± 3.65	3.67 ± 1.59	-0.66 ± 0.37	-19.45 ± 1.15	33.44 ± 3.79	-46.91 ± 2.38
PIP	0–10	-53.51 ± 5.48	-4.13 ± 2.20	2.10 ± 0.70	-0.01 ± 0.01	-25.02 ± 1.88	17.92 ± 1.81	-42.53 ± 3.04
	90–100	-59.31 ± 2.64	-6.25 ± 1.32	2.08 ± 0.47	-0.01 ± 0.03	-26.18 ± 0.98	17.98 ± 1.79	-45.75 ± 1.50
LYO-3	0–10	-64.62 ± 5.96	-39.90 ± 7.17	8.58 ± 3.18	-5.17 ± 0.88	-23.84 ± 1.80	53.13 ± 4.53	-55.55 ± 2.96
	90–100	-59.15 ± 4.69	-27.26 ± 4.05	3.47 ± 1.40	-2.33 ± 0.34	-28.00 ± 1.57	47.29 ± 3.94	-56.26 ± 5.96
BER	0–10	-81.06 ± 5.18	-7.61 ± 11.67	5.97 ± 2.34	-1.66 ± 0.25	-25.44 ± 1.97	18.21 ± 11.14	-68.44 ± 3.31
	90–100	-70.01 ± 3.02	1.98 ± 10.39	3.31 ± 1.76	-1.56 ± 0.14	-21.95 ± 0.76	8.61 ± 10.21	-59.64 ± 1.96

ΔG bind – binding free energy, Coulomb - coulomb energy, Covalent - covalent binding energy, VdW - Van der Waals energy, Lipo - lipophilic energy, Solv_GB - Generalized Born electrostatic solvation energy, H-bond - hydrogen-bonding energy. All energies are in kcal/mol.

Collectively, the *in vitro*, MD, and MM-GBSA findings imply that RES and PIP are the most promising potential inhibitors of MSMEG_5187. While studies have already established that PIP inhibits MSMEG_5187, the EP target of RES in mycobacteria has not yet been elucidated ( Huang *et al.*, 2013; Shaheen *et al.*, 2019a). Therefore, additional enzymatic assays are warranted to confirm whether RES inhibits MSMEG_5187 as suggested by the computational results. 

## Conclusion

This study has demonstrated that compounds that are either analogues of known EIs or isolated from similar plants as the known EIs putatively disrupt the efflux of BDQ, CFZ and DOX in *Msm*. While the potential EI activity of RES, BER, and PIP has previously been reported, to the best of our knowledge, this is the first study reporting the probable *mycobacteria* EI activity of LYO and LYO-3. We are currently screening BER, RES, PIP, LYO, and LYO-3 in combination with anti-TB drugs against wild type and resistant *Mtb* strains with the aim of identifying antimycobacterial drug pairs for further development.

## Declarations

### Underlying data

Zenodo: Potentiation of the antimycobacterial activity of bedaquiline, clofazimine, and doxycycline against *Mycobacterium smegmatis* by several natural product-based compounds is putatively via efflux inhibition. https://doi.org/10.5281/zenodo.17163521 ( Chacha, 2025)

The project contains the following underlying data provided as a single Excel workbook:

- Msm experiments_Robi.xlsx (Raw assay data: Concentration–response values from *in vitro* antimycobacterial assays of natural product-based compounds in combination with bedaquiline, clofazimine, and doxycycline. Includes replicate measurements, mean values, standard deviations, and IC _50_ calculations)
- Msm experiments_Robi.xlsx (Graph source data: Values underlying all figures and tables presented in the manuscript)
- Msm experiments_Robi.xlsx (Variable descriptions: Information on compound names, concentrations, replicate numbers, assay readouts, and control conditions)

Data are available under the terms of the Creative Commons Attribution 4.0 International license (CC-BY 4.0). 
