# Supplementary material for: Potentiation of the antimycobacterial activity of bedaquiline, clofazimine, and doxycycline against Mycobacterium smegmatis by several natural product-based compounds is putatively via efflux inhibition
Source: Open Res Afr. 2026 Feb 22;8:23. Originally published 2025 Oct 29. [Version 2] doi: 10.12688/openresafrica.16071.2 (PMC13077286; doi:10.12688/openresafrica.16071.2)
Supplement: Peer review history

# Reviewer report: Reviewer response for version 2

**DOI:** 10.21956/openresafrica.17627.r33834

**Published:** 2026-04-13

**Authors:** Milagros Zavaleta

**Affiliations:** 1 Research and developmentBTS Consultores S.A.C., Lima, Peru

The article presents a relevant contribution to antimycobacterial research. The study addresses the challenge of drug-resistant tuberculosis and explores efflux pump inhibition as a strategy to enhance the activity of existing anti-tuberculosis drugs. Using *Mycobacterium smegmatis* as a surrogate for *Mycobacterium tuberculosis*, the authors evaluate whether natural product-derived compounds can potentiate the activity of Bedaquiline, Clofazimine, and Doxycycline through inhibition of bacterial efflux systems, particularly MmpL5 and MSMEG_5187, a homolog of Rv1258c. The integration of experimental and computational approaches constitutes a clear strength of the work, as antimicrobial assays, checkerboard synergy testing, cytotoxicity evaluation, molecular docking, molecular dynamics simulations, and MM-GBSA analysis are used in a complementary manner.

 The selection of compounds such as Reserpine, Berberine, and Piperine is supported by previous literature and by structural similarity to known efflux pump inhibitors. The results show synergistic antimycobacterial activity, with several compound-drug combinations producing marked reductions in MICs. These findings support the hypothesis that EI contributes to the observed potentiation, and LYO and LYO-3 emerge as promising candidate efflux inhibitors. The combined in vitro and in silico findings further suggest that disruption of EP function is a plausible mechanism underlying the enhanced drug activity, while compounds related to *Berberis* species may represent useful lead molecules for the development of new EP inhibitors. The study is further strengthened by the use of quantitative synergy metrics, the inclusion of cytotoxicity data, and the identification of novel candidate inhibitors, all of which enhance its scientific relevance.

 Recommendations that could improve the manuscript further.

- The introduction would benefit from a clearer quantitative description of the global and regional burden of MDR and XDR tuberculosis in order to better frame the clinical relevance of the work.
- In addition, the discussion and conclusion could elaborate more explicitly on how the reported findings may contribute to shortening treatment duration, improving the efficacy of current drugs, and addressing MDR/XDR TB.
- Although efflux inhibition is proposed as the main mechanism, this remains indirect and has not been experimentally confirmed; therefore, direct efflux assays or gene expression analysis, such as qPCR, would strengthen the mechanistic interpretation.
- Validation of the findings in *Mycobacterium tuberculosis*, including MDR/XDR clinical isolates, would also increase translational relevance, explain its potential impact in the findings.
- Further assessment of pharmacokinetic properties and bioavailability would be advisable, particularly for compounds that do not comply with Lipinski’s rules.
- Finally, a more explicit discussion of the limitations associated with the use of a surrogate organism.

Is the work clearly and accurately presented and does it cite the current literature?

Yes

If applicable, is the statistical analysis and its interpretation appropriate?

Yes

Are all the source data underlying the results available to ensure full reproducibility?

Yes

Is the study design appropriate and is the work technically sound?

Yes

Are the conclusions drawn adequately supported by the results?

Yes

Are sufficient details of methods and analysis provided to allow replication by others?

Yes

Reviewer Expertise:

Molecular diagnostics test for infectious diseases, M. tuberculosis MDR  patterns identification

I confirm that I have read this submission and believe that I have an appropriate level of expertise to confirm that it is of an acceptable scientific standard.

# Reviewer report: Reviewer response for version 2

**DOI:** 10.21956/openresafrica.17627.r33826

**Published:** 2026-02-25

**Authors:** Carine Sao Emani

**Affiliations:** 1 Leibniz Lung Center, Borstal, Germany

I don’t have much to add, as the authors have made a strong effort to address my previous suggestions. I have read their responses and understand their points.

 For that reason, I approve the paper.

Is the work clearly and accurately presented and does it cite the current literature?

Yes

If applicable, is the statistical analysis and its interpretation appropriate?

Not applicable

Are all the source data underlying the results available to ensure full reproducibility?

No source data required

Is the study design appropriate and is the work technically sound?

Yes

Are the conclusions drawn adequately supported by the results?

Partly

Are sufficient details of methods and analysis provided to allow replication by others?

Yes

Reviewer Expertise:

NA

I confirm that I have read this submission and believe that I have an appropriate level of expertise to confirm that it is of an acceptable scientific standard.

# Reviewer report: Reviewer response for version 1

**DOI:** 10.21956/openresafrica.17246.r33527

**Published:** 2026-01-12

**Authors:** Mustapha Belaidi

**Affiliations:** 1 University of Sciences and Technologies of Oran, Oran, Algeria

This study presents a timely and well-executed investigation into combating antimicrobial resistance in tuberculosis. The research team identified natural product-based compounds that significantly enhance the effectiveness of three anti-tuberculosis drugs against *Mycobacterium smegmatis*. Using a combination of experimental and computational approaches, they demonstrate promising synergistic effects and propose efflux pump inhibition as the underlying mechanism. The work is methodologically sound, clearly presented, and makes a valuable contribution to the field, though the mechanistic conclusions remain preliminary and require direct experimental validation.

 This is a high-quality, impactful study that makes a solid contribution to antimicrobial resistance research. It is suitable for indexing and will be of interest to researchers in TB drug discovery, efflux pump biology, and natural product pharmacology. While its central mechanistic conclusion is not yet fully proven, the work is scientifically honest, rigorously performed, and lays a clear and valuable foundation for the crucial next phase of experimental validation.

 RECOMMENDATIONS for the Autor

 1-Conduct Functional Efflux Assays: Perform ethidium bromide (or fluorescent antibiotic) accumulation/efflux assays with and without the lead compounds.

 2-Validate in *M. tuberculosis*: Test synergistic combinations in relevant M. tb strains, including MDR/XDR isolates.

 3-Explore Genetic Validation: Use knockdown or knockout strains of the target efflux pumps to confirm the mechanism.

 4-Initiate Medicinal Chemistry: Optimize the most promising leads (e.g., LYO-3) based on computational insights to improve potency and reduce toxicity.

 Are the conclusions drawn adequately supported by the results? “partly”

**Answer:**The results fully support the conclusions regarding what happens (synergy, cytotoxicity) but provide only indirect, predictive support for why it happens (efflux inhibition). The conclusions are scientifically reasonable and valuable but should be interpreted as the foundation for the next phase of confirmatory research,The authors' conclusions are adequately supported as a coherent and likely hypothesis, but they are not definitively proven by the presented results. The study excellently achieves its apparent primary goal: identifying synergistic combinations and computationally modeling their potential mechanism.

 Regards

 Mustapha BELAIDI

Is the work clearly and accurately presented and does it cite the current literature?

Yes

If applicable, is the statistical analysis and its interpretation appropriate?

Yes

Are all the source data underlying the results available to ensure full reproducibility?

Yes

Is the study design appropriate and is the work technically sound?

Yes

Are the conclusions drawn adequately supported by the results?

Partly

Are sufficient details of methods and analysis provided to allow replication by others?

Yes

Reviewer Expertise:

Organic Chemistry ,physical Chemistry, computational and theoretical chemistry,and Synthesis,Catalysis & drug discovery

I confirm that I have read this submission and believe that I have an appropriate level of expertise to confirm that it is of an acceptable scientific standard, however I have significant reservations, as outlined above.

# Response

**Published:** 2026-02-07

**Authors:** ROBI CHACHA

**Affiliations:** Centre for Respiratory Diseases Research, Kenya Medical Research Institute, Nairobi, Nairobi County, Kenya

**Manuscript response to reviewers**

1. R-1: Conduct Functional Efflux Assays: Perform ethidium bromide (or fluorescent antibiotic) accumulation/efflux assays with and without the lead compounds.

R-2: Can you evaluate the effect of LYO and LYO-3 on efflux activities (with and without the tested antibiotics) using the method based on Ethidium Bromide or Nile Red, or 1,2′-Dinaphthylamine or Doxorubicin or the ColorFlux kit? **Response:** We acknowledge the importance of direct functional efflux assays (e.g., ethidium bromide, Nile Red, 1,2′-dinaphthylamine, doxorubicin, or ColorFlux-based methods) for mechanistic confirmation of efflux inhibition. However, setting up these assays is **not feasible within the scope of the present study** due to constrained funding and the fixed timelines of the Master’s training period under which the work was conducted. This limitation has now been **explicitly stated in the revised manuscript**. There are **efforts to secure additional funding** that will enable the inclusion of these functional efflux assays in subsequent studies to further substantiate the findings.  

1. Validate in *M. tuberculosis*: Test synergistic combinations in relevant *Mtb* strains, including MDR/XDR isolates.

 
**Response:** We recognize the importance of validating synergistic combinations in *Mycobacterium tuberculosis*, including MDR/XDR isolates. However, it is challenging to do these experiments within the current study due to funding limitations, biosafety requirements, and the fixed timelines of the Master’s training framework. This has now been **clearly stated as a study limitation** in the revised manuscript. **Additional funding is currently being sought** to support validation in *M. tuberculosis*, including MDR/XDR clinical isolates, in future studies.  

1. Explore Genetic Validation: Use knockdown or knockout strains of the target efflux pumps to confirm the mechanism.

 
**Response**: Genetic confirmation through targeted knockdown or knockout of candidate efflux pump genes would provide definitive mechanistic evidence. Such approaches, however, involve considerable technical complexity, extended development timelines, additional funding and specialized infrastructure that we currently are unable to explore within this study. This consideration has now been **integrated into the limitations section** of the manuscript. Future studies, contingent on **additional funding currently being pursued** **,** will incorporate genetic validation to further substantiate the proposed mechanism.  

1. Initiate Medicinal Chemistry: Optimize the most promising leads (e.g., LYO-3) based on computational insights to improve potency and reduce toxicity.

 
**Response**: We agree that medicinal chemistry optimisation of the most promising lead compounds, particularly LYO-3, guided by computational insights, would be a logical and impactful progression of this work. However, such iterative structure-activity optimisation and toxicity refinement require dedicated chemistry resources, extended timelines, and substantial financial investment that are not currently available for this study. This aspect has now been **identified as a future research direction** in the revised manuscript. **Funding and potential collaborators are currently being sought** to support medicinal chemistry-driven optimisation of lead compounds in subsequent studies.  

1. In the introduction, an information is missing in this line, after and ‘’ linezolid, and (Saukkonen et al., 2025; Singh et al., 2020).’’_

**Response:** The missing information has been added.

1. Can you perform an RT-PCR quantification of the expression of the genes encoding *MSMEG_5187* and MmpL5 when *Msm* is treated with either LYO or LYO-3?

**Response:** Quantitative RT-PCR analysis of *MSMEG_5187* and *mmpL5* expression following treatment with LYO or LYO-3 would provide complementary molecular evidence supporting efflux modulation. While technically feasible, these experiments cannot be completed within the current study due to resource limitations and competing timeline constraints associated with the training framework. This has now been **explicitly acknowledged as a study limitation** **.** **Ongoing efforts to secure additional funding** will enable targeted transcriptional analyses to directly assess efflux pump regulation in future work.

1. Can you also provide the MIC values in Molar concentrations?

**Response**: all the values have been converted to µM

1. Have you searched through the literature to find out if an ∆ *MSMEG_5187* *Msm* mutant has been generated and characterized before? If yes, what was the phenotype of this mutant? Was it sensitive to antibiotics?

**Response:** A thorough literature search was conducted and did not find any published studies reporting the generation or phenotypic characterization of an ∆ *MSMEG_5187* mutant in *Mycobacterium smegmatis*. While *MSMEG_5187* is annotated as a putative TetV-like efflux pump and has been implicated indirectly through transcriptomic studies of regulatory mutants, there are no reports describing its deletion or associated antibiotic susceptibility phenotype. Therefore, the antibiotic sensitivity of an ∆ *MSMEG_5187* mutant has not been previously documented.

1. Can you improve the resolution of Figure 3?

**Response:** This has been improved.

# Reviewer report: Reviewer response for version 1

**DOI:** 10.21956/openresafrica.17246.r33122

**Published:** 2025-12-24

**Authors:** Carine Sao Emani

**Affiliations:** 1 Leibniz Lung Center, Borstal, Germany

The authors used published data to identify putative efflux pumps inhibitors that were either structurally similar or isolated from the same source of known efflux pumps inhibitors.  Based on the obtained information, they could test a few and show that LYO or LYO-3 can also enhance the activity of known TB antibiotics. They further performed *in silico* studies to evaluate their putative interactions with known specific efflux pumps (MSMEG_5187 and MmpL5). While the manuscript is well-written and easy to follow, it would be nice if authors would include at least one *in vitro* data showing the effect of these efflux pumps inhibitor on efflux activity, since their discussion is a bit too speculative at it stands. Therefore, below are my suggestions.

  

1. In the introduction, an information is missing in this line, after and ‘’ linezolid, and (Saukkonen et al., 2025; Singh et al., 2020).’’
2. Can you perform an RT-PCR quantification of the expression of the genes encoding MSMEG_5187 and MmpL5, when Msm is treated with either LYO or LYO-3?
3. Can you also provide the MIC values in Molar concentrations?
4. Can you evaluate the effect of LYO and LYO-3 on efflux activities (with and without the tested antibiotics) using the method based on Ethidium Bromide or Nile Red, or 1,2′-Dinaphthylamine or Doxorubicin or the ColorFlux kit?
5. Have you searched through the literature to find out if an ∆ *MSMEG_5187* Msm mutant has been generated and characterized before? If yes, what was the phenotype of this mutant, was it sensitive to antibiotics?
6. Can you improve the resolution of Figure.3?

Is the work clearly and accurately presented and does it cite the current literature?

Yes

If applicable, is the statistical analysis and its interpretation appropriate?

Not applicable

Are all the source data underlying the results available to ensure full reproducibility?

No source data required

Is the study design appropriate and is the work technically sound?

Yes

Are the conclusions drawn adequately supported by the results?

Partly

Are sufficient details of methods and analysis provided to allow replication by others?

Yes

Reviewer Expertise:

Mycobacterium tuberculosis, drug targets identification and validation, drugs screening and mechanistic studies, Investigation of physiological roles of specific Mycobacterium tuberculosis enzymes

I confirm that I have read this submission and believe that I have an appropriate level of expertise to confirm that it is of an acceptable scientific standard, however I have significant reservations, as outlined above.

# Response

**Published:** 2026-02-07

**Authors:** ROBI CHACHA

**Affiliations:** Centre for Respiratory Diseases Research, Kenya Medical Research Institute, Nairobi, Nairobi County, Kenya

**Manuscript response to reviewers**

1. R-1: Conduct Functional Efflux Assays: Perform ethidium bromide (or fluorescent antibiotic) accumulation/efflux assays with and without the lead compounds.

R-2: Can you evaluate the effect of LYO and LYO-3 on efflux activities (with and without the tested antibiotics) using the method based on Ethidium Bromide or Nile Red, or 1,2′-Dinaphthylamine or Doxorubicin or the ColorFlux kit? **Response:** We acknowledge the importance of direct functional efflux assays (e.g., ethidium bromide, Nile Red, 1,2′-dinaphthylamine, doxorubicin, or ColorFlux-based methods) for mechanistic confirmation of efflux inhibition. However, setting up these assays is **not feasible within the scope of the present study** due to constrained funding and the fixed timelines of the Master’s training period under which the work was conducted. This limitation has now been **explicitly stated in the revised manuscript**. There are **efforts to secure additional funding** that will enable the inclusion of these functional efflux assays in subsequent studies to further substantiate the findings.  

1. Validate in *M. tuberculosis*: Test synergistic combinations in relevant *Mtb* strains, including MDR/XDR isolates.

 
**Response:** We recognize the importance of validating synergistic combinations in *Mycobacterium tuberculosis*, including MDR/XDR isolates. However, it is challenging to do these experiments within the current study due to funding limitations, biosafety requirements, and the fixed timelines of the Master’s training framework. This has now been **clearly stated as a study limitation** in the revised manuscript. **Additional funding is currently being sought** to support validation in *M. tuberculosis*, including MDR/XDR clinical isolates, in future studies.  

1. Explore Genetic Validation: Use knockdown or knockout strains of the target efflux pumps to confirm the mechanism.

 
**Response**: Genetic confirmation through targeted knockdown or knockout of candidate efflux pump genes would provide definitive mechanistic evidence. Such approaches, however, involve considerable technical complexity, extended development timelines, additional funding, and specialized infrastructure that we currently are unable to explore within this study. This consideration has now been **integrated into the limitations section** of the manuscript. Future studies, contingent on **additional funding currently being pursued** **,** will incorporate genetic validation to further substantiate the proposed mechanism.  

1. Initiate Medicinal Chemistry: Optimize the most promising leads (e.g., LYO-3) based on computational insights to improve potency and reduce toxicity.

 
**Response**: We agree that medicinal chemistry optimisation of the most promising lead compounds, particularly LYO-3, guided by computational insights, would be a logical and impactful progression of this work. However, such iterative structure-activity optimisation and toxicity refinement require dedicated chemistry resources, extended timelines, and substantial financial investment that are not currently available for this study. This aspect has now been **identified as a future research direction** in the revised manuscript. **Funding and potential collaborators are currently being sought** to support medicinal chemistry-driven optimisation of lead compounds in subsequent studies.  

1. In the introduction, an information is missing in this line, after and ‘’ linezolid, and (Saukkonen et al., 2025; Singh et al., 2020).’’_

**Response:** The missing information has been added.

1. Can you perform an RT-PCR quantification of the expression of the genes encoding *MSMEG_5187* and MmpL5, when *Msm* is treated with either LYO or LYO-3?

**Response:** Quantitative RT-PCR analysis of *MSMEG_5187* and *mmpL5* expression following treatment with LYO or LYO-3 would provide complementary molecular evidence supporting efflux modulation. While technically feasible, these experiments cannot be completed within the current study due to resource limitations and competing timeline constraints associated with the training framework. This has now been **explicitly acknowledged as a study limitation** **.** **Ongoing efforts to secure additional funding** will enable targeted transcriptional analyses to directly assess efflux pump regulation in future work.

1. Can you also provide the MIC values in Molar concentrations?

**Response**: all the values have been converted to µM

1. Have you searched through the literature to find out if an ∆ *MSMEG_5187* *Msm* mutant has been generated and characterized before? If yes, what was the phenotype of this mutant, was it sensitive to antibiotics?

**Response:** A thorough literature search was conducted and did not find any published studies reporting the generation or phenotypic characterization of a ∆ *MSMEG_5187* mutant in *Mycobacterium smegmatis*. While *MSMEG_5187* is annotated as a putative TetV-like efflux pump and has been implicated indirectly through transcriptomic studies of regulatory mutants, there are no reports describing its deletion or associated antibiotic susceptibility phenotype. Therefore, the antibiotic sensitivity of an ∆ *MSMEG_5187* mutant has not been previously documented.

1. Can you improve the resolution of Figure 3?

**Response:** This has been improved
